# Wearable-measured heart rate variability and premenstrual disorder symptoms across menstrual cycle

**DOI:** 10.1007/s00737-026-01740-z

**Published:** 2026-07-07

**Authors:** Qing Pan, Jing Zhou, Min Chen, Peijie Zhang, Xinyi Shi, Yifei Lin, Jin Huang, Yuchen Li, Donghao Lu

**Affiliations:** 1 Health Management Center, General Practice Medical Center, Innovation Institute for Integration of Medicine and Engineering, West China Hospital, Sichuan University, Chengdu, China; 2 Institute of Environmental Medicine, Karolinska Institutet, Stockholm, Sweden; 3 Mental Health Center, West China Hospital, Sichuan University, Chengdu, China; 4 Department of Nutrition and Food Hygiene, West China School of Public Health and West China Fourth Hospital, Sichuan University, Chengdu, Sichuan China

**Keywords:** Premenstrual syndrome, Premenstrual dysphoric disorder, Wearable device, Heart rate variability, Women’s health, Menstrual cycle

## Abstract

**Background:**

Screening premenstrual disorders (PMDs) is time-consuming and challenging, resulting in delays in detection and treatment. To characterize the temporal dynamics of portable digital-marker across the menstrual cycle and its biological basis in PMDs, we investigated the menstrual fluctuation of wearable device-based real-time heart rate variability (HRV) through menstrual cycles and its associations with premenstrual disorders (PMDs) symptoms.

**Methods:**

A prospective study of female participants nested from the Care of Premenstrual Emotion Cohort was conducted. Daily HRV metrics were collected by the Huawei Fitness Tracker over 1–2 menstrual cycles. PMDs symptoms were assessed with the Daily Record of Severity of Problems on a daily basis. HRV variability across cycles was described using descriptive statistics and splines, while associations between HRV metrics and PMDs symptoms were estimated using a mixed-effect model.

**Results:**

In total, 193 participants (with 68 prospectively confirmed PMDs) were included, with measures from 293 menstrual cycles. In both women with and without PMDs, SDNN, rMSSD, and HF decreased before menses and increased afterwards; the increase trends were more pronounced in women without PMDs. During the week before or after menses, levels of these HRV metrics were inversely associated with PMDs symptoms among women with PMDs (e.g., rMSSD, postmenstrual week, β = -0·036 per SD, 95% CI: -0·065 to -0·048), whereas null association was noted for those without PMDs (β = -0·001, 95% CI -0·011 to 0·009; *P*-for-difference < 0·001).

**Conclusions:**

Wearable device-estimated HRV fluctuate across menstrual cycles, with varying strengths of association with PMDs symptoms between individuals with and without PMDs, which may facilitate the development of digital biomarkers for future PMDs diagnostics.

**Supplementary Information:**

The online version contains supplementary material available at https://doi.org/10.1007/s00737-026-01740-z.

## Introduction

Premenstrual syndrome (PMS) and premenstrual dysphoric disorder (PMDD), together coined as premenstrual disorders (PMDs), affect about 20 to 30% and 2 to 8% women of reproductive age, respectively (Yonkers and Simoni 2018). These patients typically suffer from various affective and physical symptoms before menses, entailing functional impairments on social, relationship, and work performance (Comasco et al. 2021, Abbas et al. 2020). Our recent studies have illustrated a range of health consequences following PMDs, such as peripartum depression (Yang et al. 2024), menopause symptoms (Yang et al. 2023), suicidal behavior (Yang et al. 2022), and premature death for those diagnosed in young ages (Opatowski et al. 2024). It may also contribute to the well-documented sex-disparity in mental health (Li et al. 2023).

Despite the high prevalence and tremendous impact, clinical challenges prevent these patients from early detection and effective treatment. On average, it takes the patients six visits to different healthcare professionals and a staggering 12 years to receive a diagnosis for PMDD (International ). In addition to the limited awareness of PMDs among healthcare providers, the sole diagnostic tool involves maintaining a daily symptom chart for two menstrual cycles before receiving treatment. It is exceedingly time-consuming and challenging for both patients (who must diligently complete questions over two months) and clinicians (who must review extensive longitudinal data). To streamline and expedite the diagnostic process for PMDs, there is an urgent need for a diagnostic tool that utilizes objective and easily accessible measures.

Heart rate variability (HRV) has been established as a reliable, non-invasive indicator of autonomic nerves system (ANS) activity (Fan et al. 2024), and has been linked to several core symptoms of PMDs, e.g., anxiety, depression, and irritability (Hartmann et al. 2018, Cheng et al. 2022, Naim et al. 2021). Among healthy women, a decrease in HRV has been consistently observed during late luteal phase (Brar et al. 2015, Yildirir et al. 2001, Yazar and Yazıcı 2016, Pestana et al. 2018). Moreover, a recent large-scale study using wearable devices illustrated that HRV fluctuated in a regular pattern across the menstrual cycle (Jasinski et al. 2024), althoupredictive gh it is unclear how the menstrual cycle was reported by the participants (retrospectively recalled or prospectively reported). Moreover, the association between HRV and PMDs symptoms, as well as whether HRV patterns differ between women with and without PMDs, remains unknown. To date, only a few studies base on small sample size (14–29 cases (Landén et al. 2004, Baker et al. 2008, Zambotti et al. 2013, Kulshreshtha et al. 2013)) and varying, yet up to 24-h, recording length have found differences in HRV between women with and without PMDs (Landén et al. 2004). While these findings are proactive, longitudinal measurement of HRV is needed to evaluate its potential for monitoring and diagnosing PMDs. Consumer wearables are widely used in young people and have emerged as a convenient and reliable tool for collecting real-time HRV (Fan et al. 2024, Georgiou et al. 2018). Leveraging real-time HRV from a wearable device at a minute-scale alongside with daily reported menstrual cycle data, we aimed to characterize the temporal patterns of HRV across the menstrual cycle, and assess the association of HRV with PMDs symptoms between individuals with and without PMDs.

## Methods

### Study design

The Care of Premenstrual Emotion (COPE) study is a prospective cohort of medical students at Sichuan University initiated in 2021. It aimed to collect information on demographics, health behaviors, bio-samples, and wearable device-based biomarkers of PMDs (Shi et al. 2024). A total of 1931 female students aged 18–32 from West China Schools of Medicine (including Clinical Medicine, Stomatology, Basic Medical Sciences & Forensic Medicine, Public Health, and Pharmacy) were recruited. The COPE study was approved by the Institutional Review Board of West China Hospital, Sichuan University (No. 2023 − 179). All participants were fully informed about the purpose and content of the study and provided their electronic consent to participate.

### Participants

The present study was based on a sub-population of natural cyclers from the COPE cohort. Participants were initially screened for PMDs using a modified version of the Calendar of Premenstrual Symptoms scale. This scale includes 8 affective and 19 physical/behavioral symptom items, followed by 3 functional impact items, all measured on a 4-point Likert scale. The classification criteria have been described in our previous study (Shi et al. 2024). All participants who screened positive for PMDs were invited, while a random sample of those who screened negative were recruited (Supplementary Methods). They were asked to wear the tracker for at least one cycle, and complete prospective symptom charting for two cycles (one cycle for those screened negative). Individuals with severe somatic diseases or neurological disorders were not invited. After excluding 9 withdrawals and 2 with a high missing rate on prospective symptom charting, a total of 193 participants were included for analysis. Such differential sampling resulted in a higher proportion of PMDs in the present analytic sample than in the full COPE cohort; inverse-probability weights were applied in all analyses to restore representativeness (see Statistical Analysis).

### HRV measurements

All participants were instructed to wear a Huawei Fitness Tracker 6 Pro for at least 12 h each day throughout their menstrual cycles and were required to upload their daily records to a cloud-based service. The fitness tracker is a wristband equipped with photoplethysmography (PPG) sensor, it automatically collects real-time metrics, including RRI, heart rate, skin temperature, blood oxygen saturation, physical activity, and sleep episodes. The validity of PPG-derived HRV has been evaluated against Holter ECG in previous studies. In a 24‑hour simultaneous recording study involving 159 participants, HRV indices derived from nocturnal PPG and ECG readouts showed concordance during artifact‑free sleep phases, with correlation coefficients between the two modalities ranging from 0.28 to 0.87 ^10^. In a 72‑hour prospective study of 50 high‑risk patients, quality‑controlled wrist‑based PPG signals demonstrated a sensitivity of 81.9% and a specificity of 96.6% relative to Holter ECG (Guo et al. 2021).

To mitigate signal noise and motion artifacts inherent to wrist-based PPG, a four-layer quality control protocol was implemented. First, HRV metrics were derived from resting-state PPG records averaged across the 3:00–5:00 a.m. window, which coincides with the sleep phase when participant movement is naturally minimal.Second, device-level signal quality filtering was applied. The Huawei fitness tracker provides a beat-to-beat signal quality index (SQI) ranging from 0 to 100, embedded in the raw PPG output. Individual beats with an SQI below 85 were excluded, thereby removing epochs contaminated by motion-induced or electronic noise prior to HRV calculation.Third, physiological plausibility filtering was performed at the single-beat level. RRI values outside the 300–2,000 ms range (corresponding to heart rates of 30–200 bpm) were excluded as noise-induced outliers or ectopic beats. Fourth, adaptive outlier removal was conducted on the non-interpolated heart rate (NIHR) series using the FilterNIHR function from the RHRV package. This function adaptively detects ectopic beats (premature atrial and ventricular contractions) and high-frequency signal artifacts by comparing beat-to-beat interval changes against a moving-average threshold with standard deviation–based bounds, thereby converting the raw RRI sequence into a cleaned normal-to-normal (NN) interval series.

Then five most commonly used HRV metrics were calculated (Jasinski et al. 2024, Kayacan et al. 2021), including two time-domain and three frequency-domain parameters. The two time-domain parameters comprised SDNN (Standard Deviation of NN intervals) and rMSSD (Root Mean Square of Successive Differences between NN intervals). To compute frequency-domain metrics, the original non-uniformly distributed RRI records were further transformed into uniformly distributed records at 4 Hz (four records per minute). This transformation employs linear interpolation to estimate RRI values at uniformly distributed time points. Subsequently, three frequency-domain parameters, including LF (absolute power in the low-frequency band, 0·04–0·15 Hz), HF (absolute power in the high-frequency band, 0·15–0·4 Hz), and LF/HF (the ratio of LF to HF) were calculated by Fourier transformation.

### Assessment of PMDs symptoms

Participants were asked to complete a questionnaire daily for two consecutive menstrual cycles concerning PMDs symptoms and record menstruation occurrence. PMDs symptoms were assessed with a Chinese version (Wu et al. 2013) of Daily Record of Severity of Problems (DRSP) (Endicott et al. 2006), which has been proven to be both sensitive and reliable in measuring symptoms and impairment for PMDs (Endicott et al. 2006). The DRSP consists of 24 items, each rated on a 6-point Likert scale ranging from 1 (not at all) to 6 (extreme). For each individual item, the average values of before (day − 5 to -1) and after (day 6 to 10) menses were calculated separately for each cycle to estimate the symptom change from pre- to post-menstrual period. In line with established criteria (Feingold et al. , Association 2022), individuals were diagnosed with PMDs if they met the following criteria be met independently in both consecutive cycles: (1) at least one core symptom scores > 2 on average in premenstrual days ( Day − 5 to -1) and with 20% decrease in postmenstrual days (Day 6 to 10); (2) in addition, at least four symptom scores > 1 in premenstrual days and with 20% decrease in postmenstrual days. (3) at least one functional impairment scores (work/school efficiency, home responsibilities, or social/leisure activities) > 2 in premenstrual days. Among the PMDs cases, individuals were further classified as PMS and PMDD; the latter was defined as (1) at least one core symptom scores > 3 on average in premenstrual days and with 20% decrease in postmenstrual days; (2) at least four symptom scores > 2 in premenstrual days and with 20% decrease in postmenstrual days. (3) a least one functional impairment scores > 3 in premenstrual days. The definitions of core symptoms were listed in Supplement Methods.

### Covariates

Participants provided information on their age and schools at recruitment, and risk factors of PMDs, e.g., age of menarche, body mass index (BMI; calculated from self-reported height and weight), and alcohol consumption (having consumed alcohol at least once, or heavy drinking, defined as consuming five or more drinks within one to two hours, during the past 30 days). Smoking status was not included as a covariate due to its low prevalence (1.85%) among young women in China (Xia et al. 2024).

### Statistical analysis

Because all participants who screened positive for PMDs were invited while only a random sample of those who screened negative were recruited, the proportion of PMDs in the analytic sample (35·2%) was higher than that in the full COPE cohort (23·4%). To restore representativeness of the source cohort, inverse-probability weights were calculated as the inverse of the selection probability for each group. Specifically, individuals who screened positive were assigned a weight of 0·664 (0·234/0·352), and those who screened negative were assigned a weight of 1·182 ((1 − 0·234)/(1 − 0·352)). These weights were incorporated into all mixed-effects models.

We estimated the average levels of the five HRV metrics (including mean and standard deviation for normally distributed parameters and the median and percentiles for non-normally distributed parameters) by days across menstrual cycle among individuals with and without PMDs. To visualize the daily fluctuations, we applied natural cubic splines with two knots at day − 5 and day 8, spaced at 33% and 67% quantiles of menstrual days. Moreover, we employed a mixed effect model to estimate the weekly association between HRV and PMDs symptoms, because both HRV metrics and PMDs symptoms were repeatedly measured. All the mixed-effects models were adjusted for the above mentioned physiological and behavioral covariates. The weeks were defined as two weeks before (days − 14 to -8), one week before (days − 7 to -1), one week after (days 1 to 7), and two weeks after menses (days 7 to 14). *Z* score test was used to compare the association strength between individuals with and without PMDs across menstrual weeks.

To illustrate the relationship with different PMDs symptom domains, we assessed the associations of HRV metrics with physiological/behavioral and affective symptom scores (z-score), separately. Definitions of the physiological/behavioral and affective symptom scores can be found in the Supplement Methods. To shed light on the disease severity, we also assessed the association between HRV and PMDs symptoms between PMDD versus PMS. Since our samples included sub-clinical PMDs (assessed as controls), we conducted a sensitivity analysis restricting the controls to “pure controls” (i.e., those with minimum PMDs symptoms). The pure controls were defined as reporting ‘not at all’ or ‘minimal’ for all individual symptoms and the impact questions. Last, sensitivity analysis was performed in subpopulations reporting regular menstrual cycles with a length of 26 to 31 days.

All analyses were conducted using R statistical software (Version 4·3·2; R Core Team 2023), with significance set at α = 0·05. A *P*-value less than or equal to 0·05 was considered statistically significant.

## Results

### Characteristics

A total of 193 participants with daily records across 293 menstrual cycles were included in analysis, with an average age of 19·92 ± 1·65 years. Among them, 68 (35·2%) were prospectively diagnosed with PMDs, including 41 (21·2%) with PMS and 27 (14·0%) with PMDD. Individuals with PMDs were statistically comparable to those without regarding age, age at menarche, BMI, and alcohol consumption (all *P* > 0·05; Table 1).

**Table 1:** Characteristics between individuals with PMDs and without PMDs

Characteristics	Without PMDs (*N* = 125)	PMDs (*N* = 68)	*P* value
Age (years)	19·82 ± 1·41	20·09 ± 2·00	0·28
Age at menarche (years)	12·34 ± 1·22	12·31 ± 1·25	0·86
BMI (kg/m^2^)	21·14 ± 4·59	20·79 ± 2·43	0·56
Drinking during the past 30 days			0·24
0 day	57 (46)	23 (34)	
1-2days	26 (21)	19 (28)	
> 3 days	41 (33)	26 (38)	
Drinking heavily during the past 30 days			0·46
0 day	76 (61)	44 (65)	
1-2days	14 (11)	4 (6)	
> 3 days	34 (27)	20 (29)	

PMDs: premenstrual disorders, including PMS and PMDD. BMI: Body mass index; Drinking heavily: consuming at least 5 drinks within one or two hours

### PMDs symptom and HRV levels across menstrual cycle

**Fig. 1:**
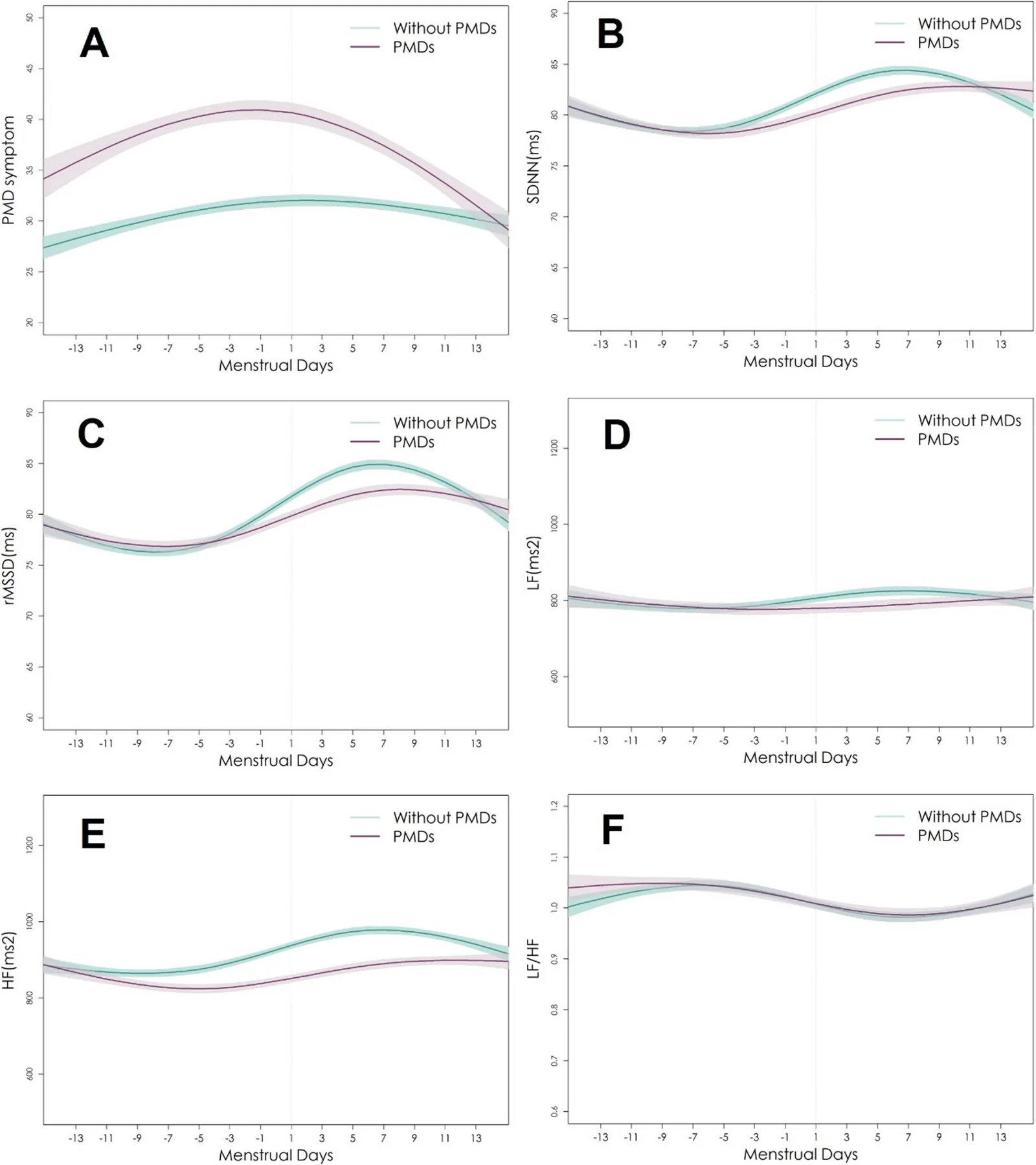
Comparisons of PMDs symptom score and HRV levels across menstrual cycle between individuals with and without PMDs. (Note: PMDs: premenstrual disorders, including PMS and PMDD. **A**: PMD symptom: Symptom scores for premenstrual disorders were assessed using the Daily Record of Severity of Problems scale; **B**: SDNN: standard deviation of RR intervals; **C**: rMSSD: Root mean square of successive RR interval differences; **D**: LF: absolute power in the LF band (0.04–0.15 Hz); **E**: HF: absolute power in the HF band (0.15–0.4 Hz); F: LF/HF: ratio of LF to HF.)

Among individuals with PMDs, a bell-shape pattern was observed for PMDs symptom across menstrual cycle, with the peak at the day before menses (Day − 1; Fig. 1A). In contrast, a relatively flat pattern with lower symptom score was found for those without PMDs. Regarding HRV, we observed a decrease of SDNN in early luteal phase (from Day − 13 to -6) and an increase from Day − 5 until Day 11 (Fig. 1B) for both individuals with and without PMDs, with a more pronounced elevation among those without PMDs. Similar patterns were found for rMSSD and HF (Fig. 1C/E). The variation pattern for LF and LF/HF ratio was largely similar between those with and without PMDs (Fig. 1D/F) .

### Associations between HRV and PMDs symptom

**Fig. 2:**
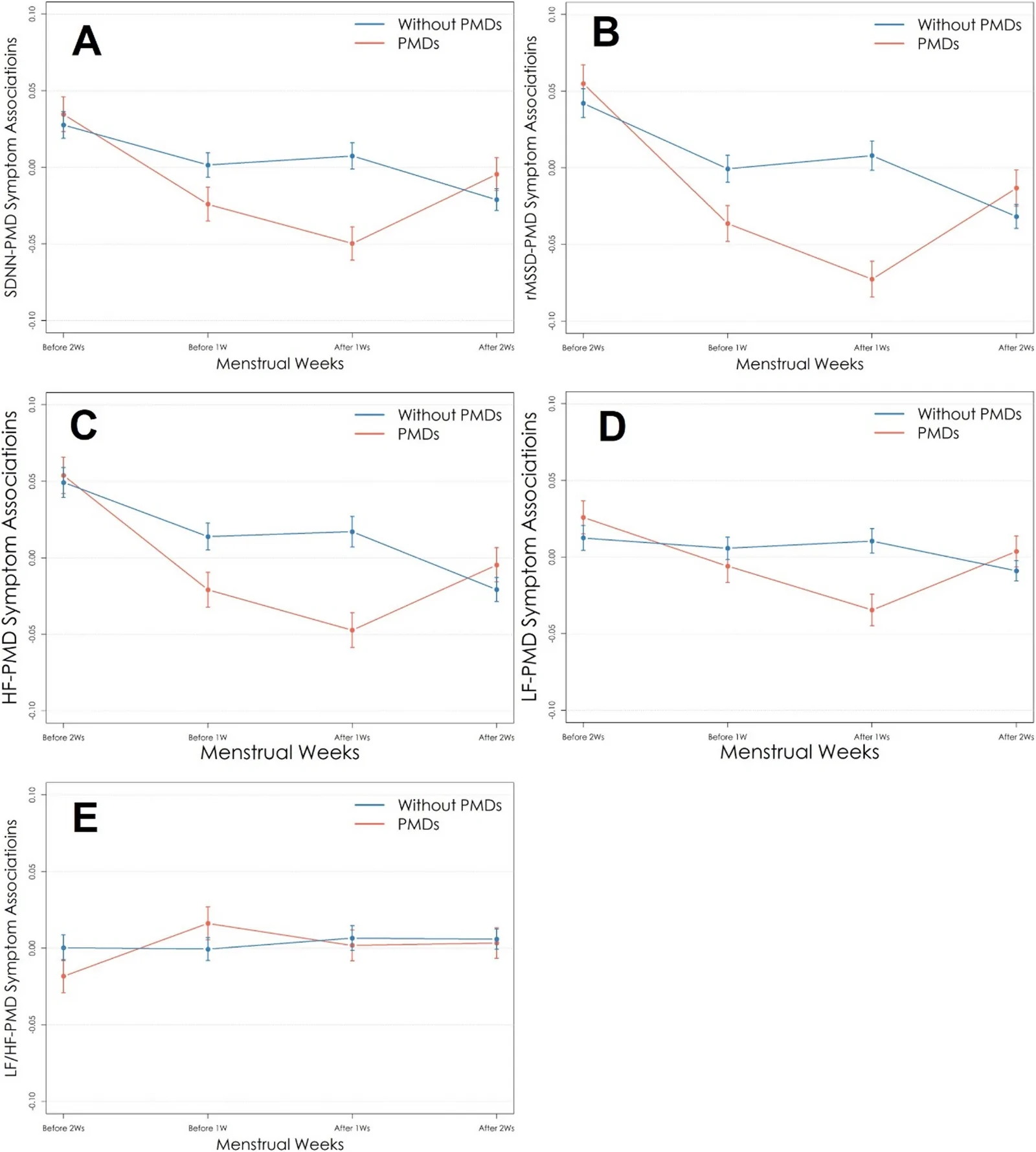
Comparisons of the associations between HRV and PMD symptoms among individuals with PMDs and without PMDs across menstrual cycle. (Note: PMDs: premenstrual disorders, including PMS and PMDD. **A**: SDNN: standard deviation of RR intervals; **B**: rMSSD: Root mean square of successive RR interval differences; **C**: HF: absolute power in the HF band (0·15–0·4 Hz); **D**: LF: absolute power in the LF band (0·04–0·15 Hz); **E**: LF/HF: ratio of LF to HF. The models have been adjusted for age, age at menarche, BMI, drinking during the past 30 days, and drinking heavily during the past 30 days.)

While we presented the associations over four weeks across menstrual cycle, we focused on results during the week before (symptom window) and the week after menses (symptom-relieving window). During the week before menses, the SDNN level was inversely associated with PMDs symptom among individuals with PMDs (β = -0·024 per SD increase, 95% CI -0·036- -0·041; Fig. 2A and point estimates in Table S1), whereas null association was noted among those without PMDs (β = 0·001, 95% CI -0·007 − 0·009; *P*-for-difference < 0·001). Even stronger association difference between those with and without PMDs was found during the week after menses (*P*-for-difference < 0·001). Largely comparable trends were also noted for rMSSD, HF, and LF (Fig. 2B, C and D) but not observed for LF/HF (Fig. 2E).

### Additional analyses

**Fig. 3:**
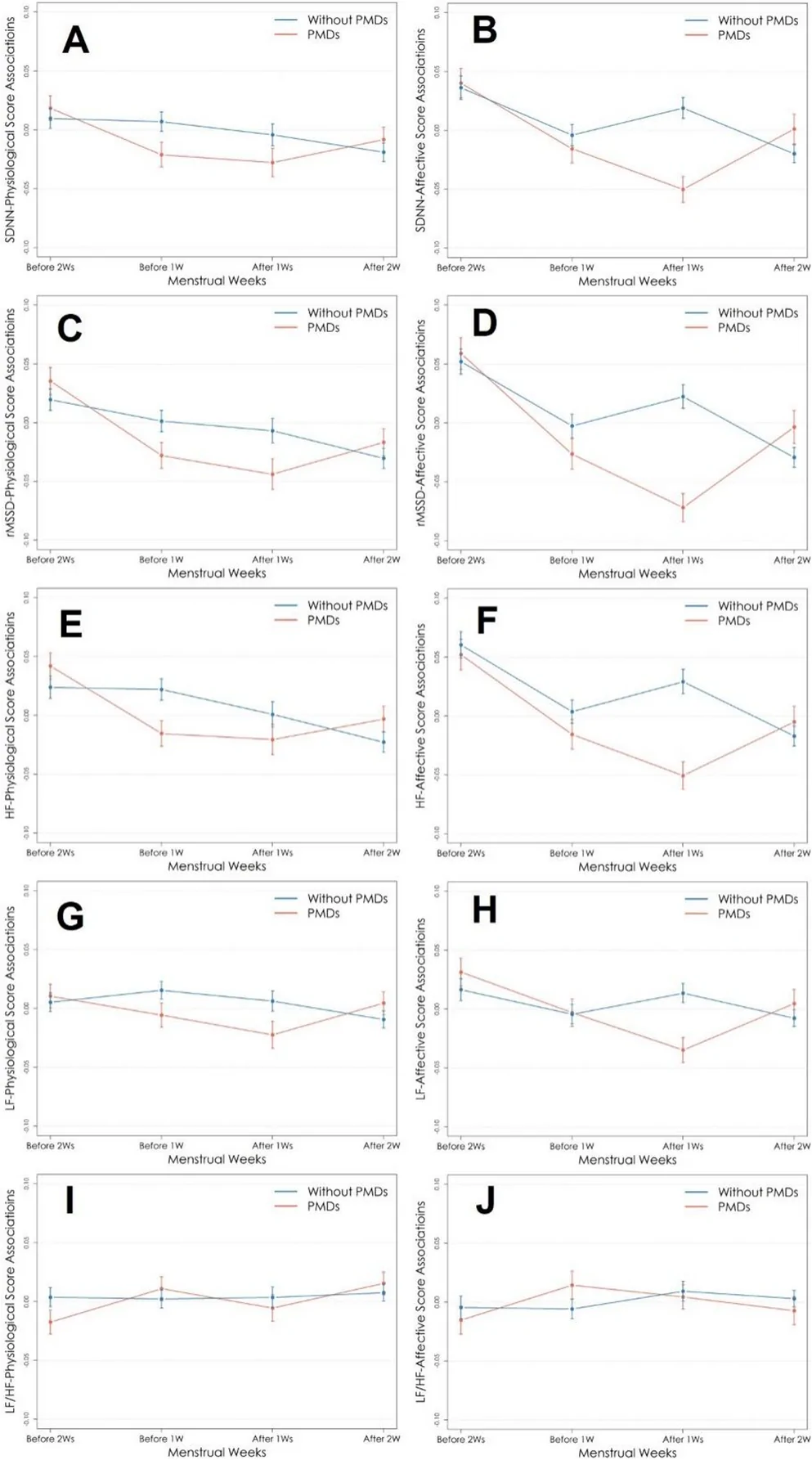
Comparisons of the associations between HRV and physiological/behavioral and affective symptoms among individuals with PMDs and without PMDs across menstrual cycle. (Note: PMDs: premenstrual disorders, including PMS and PMDD. PMDD: premenstrual dysphoric disorder; PMS: premenstrual syndrome; **A**: SDNN for physiological/behavioral sub-scores; **B**: SDNN for affective sub-scores; **C**: rMSSD for physiological/behavioral sub-scores; **D**: rMSSD for affective sub-scores; **E**: HF for physiological/behavioral sub-scores; **F**: HF for affective sub-scores; **G**: LF for physiological/behavioral sub-scores; H: LF for affective sub-scores; I: LF/HF for physiological/behavioral sub-scores; J: LF/HF for affective sub-scores. The models have been adjusted for age, age at menarche, BMI, drinking during the past 30 days, and drinking heavily during the past 30 days.)

**Fig. 4:**
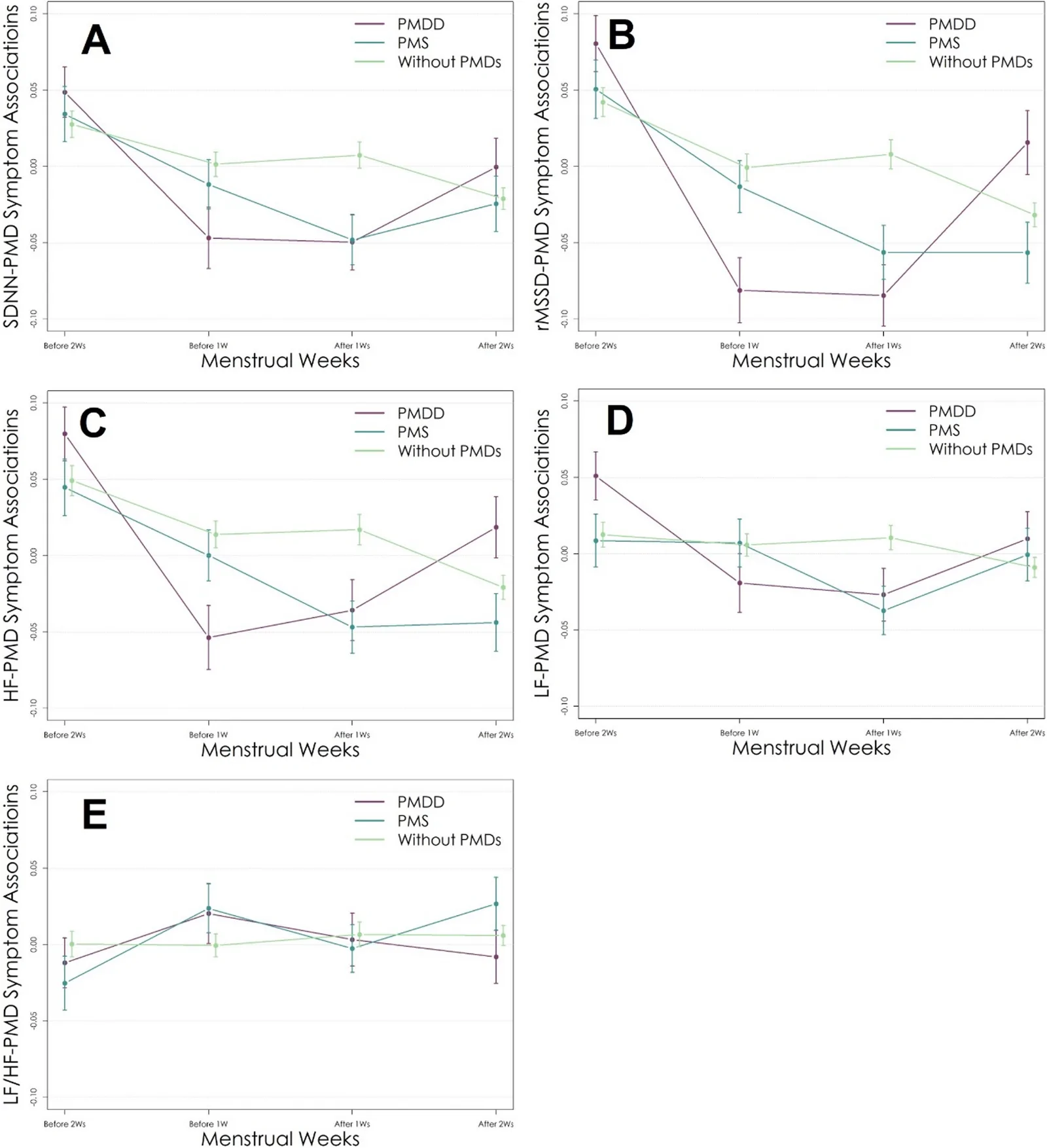
Comparison of the associations between HRV and PMD symptoms among individuals with PMDD, PMS, and without PMDs across the menstrual cycle. (Note: PMDs: premenstrual disorders, including PMS and PMDD; PMS: premenstrual syndrome; PMDD: premenstrual dysphoric disorder. Non-PMDs: individuals without PMDD or PMS. **A**: SDNN: standard deviation of RR intervals; **B**: rMSSD: Root mean square of successive RR interval differences; **C**: HF: absolute power in the HF band (0·15–0·4 Hz); **D**: LF: absolute power in the LF band (0·04–0·15 Hz); **E**: LF/HF: ratio of LF to HF. The models have been adjusted for age, age at menarche, BMI, drinking during the past 30 days, and drinking heavily during the past 30 days.)

When analyzing physiological/behavioral and affective symptoms separately, we observed similar patterns found in the primary analysis (Fig. 3 and estimates in Table S2), yet stronger difference for affective symptoms between individuals with and without PMDs. Regarding PMDs severity, we found that the inverse association of SDNN were comparable between PMS and PMDD groups during the week after menses (*P*-for-difference 0·868), while a stronger association was found for PMDD within the week before menses (*P*-for-difference 0·006). Similar heterogeneous associations were also observed for rMSSD, HF, and LF (Fig. 4 and Table S3). In addition, we compared PMDs with a pure control group consisting of 43 individuals and yielded comparable trends (Figure S1 and Table S4). Last, we restricted the analysis to individuals with regular menstrual cycles with a cycle length of 26 to 31 days and observed similar results (Figure S2-3 and Table S5).

## Discussion

To the best of our knowledge, this study is the first investigation into the real-time dynamics of HRV across menstrual cycles and its associations with PMDs symptoms. We showed that measuring with wearable device, most HRV indicators fluctuated across the menstrual cycle in both healthy women and women with PMDs, with more pronounced alteration from weeks before menses to weeks after menses among healthy women. Our findings highlight that information on menstrual cycle phase should be considered in future HRV studies involving women. Moreover, HRV was inversely associated with PMDs symptoms during the week before and after menses among women with PMDs, whereas no such link was found for healthy women. The association was particularly stronger with affective symptoms and during the premenstrual week among women with PMDD. Given the common use of wearable device and easy access to passively collected HRV readouts, our findings indicate that HRV metrics mirror symptom fluctuations in PMDs and may serve as a foundation for developing objective monitoring tools for these disorders.

### HRV across menstrual cycles

Previous studies based on single-time or short-period measurement have documented that HRV indicating parasympathetic activity (SDNN, rMSSD, and HF) decreases during luteal phase compared to follicular phase in healthy women (Brar et al. 2015, Yildirir et al. 2001, Yazar and Yazıcı 2016, Pestana et al. 2018, Schmalenberger et al. ). More recently, a large-scale study using a wrist-worn wearable device found that rMSSD reached its maximum around day 5 and minimum around day 27 of the menstrual cycle (Jasinski et al. 2024). Consistent with this general pattern, our study also observed a decrease in RMSSD prior to menstruation and an increase following the onset of menses in healthy women, although the exact timing differed slightly. Specifically, we found that rMSSD reached its lowest value on day − 2 (two days before menstruation) and peaked on day 5. This might be due to the difference in menstrual cycle reporting: to accurately record the menstrual cycle, we prospectively collected cycle information by asking participants daily, while it is unclear whether the cycle was retrospectively recalled in the other study. Furthermore, beyond rMSSD, we found that other HRV parameters reflecting parasympathetic activity, including SDNN and HF, decreased during early luteal phase, with the lowest level about one week before menses before it bounced back and peaked around one week after menses.

Additionally, the LF/HF ratio showed a slight decrease before menses, followed by an increase afterward. Notably, this temporal pattern coincided with the concurrent decrease in SDNN, rMSSD, and HF during the premenstrual phase and their subsequent increase after menstruation. This may be explained by the fact that during the premenstrual phase, as parasympathetic activity decreases, sympathetic activity gradually becomes dominant. After menstruation, parasympathetic activity regains dominance. Hormonal shifts during menstrual cycle might explain these HRV fluctuations. The rise in estrogen levels after menses enhances parasympathetic activity and suppresses sympathetic outflow, thereby contributing to elevated SDNN, rMSSD, and HF values (Vongpatanasin et al. 2001). Conversely, the peak in progesterone levels in luteal phase attenuates baroreflex sensitivity and augments sympathetic activation (Zambotti et al. 2013, Minson et al. 2000).

Limited evidence is available on HRV trajectory across menstrual cycle among individuals with PMDs. In a US study of 9 cases and 12 controls with selected two specific days from follicular and luteal phases respectively (Baker et al. 2008), lower SDNN, rMSSD, and HF values were noted during the late luteal phase among women with PMDs compared to those without, which is in line with our finding. However, we found that during most post-menstruation days (corresponding to the follicular phase in the previous study), SDNN, rMSSD, and HF remained lower in individuals with PMDs when compared with healthy controls, whereas the previous study reported these values as being higher in the PMS group compared to controls. While the discrepancy might be explained by the small sample size and limited measurement length in the previous study, future studies are warranted to confirm our findings. If replicated, our results suggest that the ability of restoring parasympathetic activity from late luteal to early follicular phase is impaired among women with PMDs.

### HRV and PMDs symptoms

To our knowledge, there are only two studies investigating the association between HRV and PMDs symptoms. In a Polish study with 177 women of reproductive age, including both patients with PMDs and healthy controls, Danel et al. found a positive relationship between PMDs symptoms and HRV indices (SDNN and rMSSD, measured within 10 min intervals) during follicular phase in women without use of hormonal contraception, but not for those with use of hormonal contraception (Danel et al. 2019). Another study tracking three participants throughout the entire menstrual cycle reported that, within individuals, log(rMSSD) (measured within 6 min intervals) was inversely correlated with psychological symptom but showed no association with physiological symptom (Blaser et al. 2023). While the majority of our participants did not use hormonal contraceptives (95·5%), our study significantly differs from previous ones by design (larger sample size and consecutive measurements on both HRV and PMDs symptoms), and unsurprisingly yielded different findings. For instance, we observed that in one week before or after menses, rMSSD levels were inversely associated with PMDs symptoms among individuals with PMDs, whereas no significant association was found for those without PMDs. The association was also observed for SNDD and HF, and it was particularly stronger during the one week after menses. Our findings on the heterogeneous association between HRV and PMDs symptoms around menses between women with and without PMDs suggest that HRV metrics may serve as physiological correlates of menstrual fluctuations, reflecting biological markers associated with symptom severity in PMDs. Furthermore, a recent scoping review of 40 studies reported that wearable photoplethysmography (PPG)-derived HRV varies across the menstrual cycle and is inversely associated with the severity of premenstrual symptoms in premenstrual syndrome (PMS) and premenstrual dysphoric disorder (PMDD), which is consistent with the findings of this study (Johnson et al. 2026).

Although the exact mechanism linking ANS activity to PMDs remains unclear, the prevailing theory supports a role of hormonal fluctuations (Barth et al. 2015, D’Souza et al. 2019). During luteal phase, fluctuations in estrogen and progesterone can disrupt ANS balance by affecting neurotransmitters such as norepinephrine and serotonin (Del Río et al. 2018). Increased sympathetic nervous activity has been associated with anxiety, irritability, and other emotional symptoms, while reduced parasympathetic activity is linked to difficulties in emotional regulation (Bellato et al. 2024). On the other hand, ANS regulates physiological functions such as blood pressure, heart rate, and HRV. Specifically, certain HRV values would increase as parasympathetic effect increases, while HRV would decrease as sympathetic effect increases (Goldberger et al. 2001). The relationship between HRV and PMDs symptoms observed in our data may therefore speak for the phenotypic link underlying ANS activity. This is further supported by our data showing that the association between HRV and affective symptoms was stronger than for physiological/behavioral symptoms.

### Strengths and limitations

The strength of this study lies in real-time monitoring of HRV, coupled with prospective PMDs symptom charting in a relatively large sample, providing a panoramic picture of autonomic function and PMDs symptoms across menstrual cycles. However, several limitations need consideration. First, the PPG sensor in the wristband may be affected by motion artifacts and variations in fit tightness, potentially leading to inaccuracy in HRV readings. However, it has been validated that HRV metrics collected by PPG sensor from Huawei fitness tracker are comparable to those obtained from medical ECGs (Fan et al. 2024).Moreover, we calculated HRV during the resting state by averaging records collected between 3:00 and 5:00 a.m., when participants are less likely to move. Second, although PMDs symptoms were assessed daily through validated electronic survey and PMDs cases were confirmed based on established criteria, self-reported symptoms may introduce inaccuracy due to variations in individual interpretation. However, the temporal pattern of PMDs symptoms were assessed within the same individual for diagnosing PMDs. In addition, our study aimed to assess contemporaneous associations between HRV and PMDs symptoms instead of claiming causal relationship, although a few known confounders were adjusted for in our analyses. Moreover, Our findings are based on female university students from health-related disciplines, potentially limiting generalizability to populations with different biological or sociodemographic characteristics underlying the relationship between HRV and PMD symptoms. However, given young adults’ widespread use of wearable devices, our results remain informative for similar populations, although replication in broader samples is needed.

Additionally, we acknowledge that categorizing menstrual days into weekly windows, while enhancing statistical stability and clinical interpretability, may obscure more nuanced, nonlinear, or phase-specific fluctuations that could be captured with daily-level modeling approaches. Future studies leveraging the richness of daily wearable data could employ these refined techniques to further clarify the temporal dynamics of HRV in relation to PMDs symptoms. Last, we acknowledge that the physiological interpretation of LF/HF ratio remains debated in the literature (Hayano and Yuda 2021). Consequently, we urge readers to interpret our primary findings through SDNN, rMSSD, and HF—metrics that are more widely accepted as indicators of parasympathetic activity and overall autonomic function—rather than relying on LF or LF/HF ratio.

## Conclusions

In summary, our findings suggest that HRV metrics fluctuate across the menstrual cycle in healthy young women, highlighting that menstrual cycle phase should be considered in future HRV studies involving young women. Our findings also illustrate that several HRV metrics are only inversely associated with PMDs symptoms during the week before and after menses among young women with PMDs. While future studies based on independent populations are needed to confirm the predictive value, these proactive findings illuminate that HRV metrics could play a crucial role in improving the screening and diagnostic process for PMDs among young women in the future.

## Supplementary Information

Below is the link to the electronic supplementary material.

Supplementary Material 1

## Data Availability

De-identified individual participant data and a data dictionary defining each field used for analysis can be made available upon approval of a research proposal to the COPE cohort study group. Contact should be made with the COPE steering group ( [copestudy@wchscu.cn](mailto: copestudy@wchscu.cn) ).

## Code availability

The scripts used to complete the primary analyses in this manuscript can be made available upon approval of a research proposal to the COPE cohort study group. Contact should be made with the COPE steering group (copestudy@wchscu.cn).

## Declarations

### **Reprints and permission information**

This manuscript has been submitted as a preprint on medRxiv at https://www.medrxiv.org/content, https://doi.org/10.1101/2024.10.27.24316196v1.

## References

[CR3] Abbas K, Usman G, Ahmed M et al (2020) Physical and psychological symptoms associated with premenstrual syndrome and their impact on the daily routine of women in a low socioeconomic status locality. Cureus 12:e10869. 10.7759/cureus.10821 PMC7645292 33173629

[CR30] American Psychiatric Association (2022) Depressive disorders. In: Diagnostic and statistical manual of mental disorders, 5th edn, text rev. American Psychiatric Association Publishing, Washington, DC. 10.1176/appi.books.9780890425787.x04_Depressive_Disorders

[CR20] Baker FC, Colrain IM, Trinder J (2008) Reduced parasympathetic activity during sleep in the symptomatic phase of severe premenstrual syndrome. J Psychosom Res 65:13–22. 10.1016/j.jpsychores.2008.04.008 18582607 PMC2519123

[CR38] Barth C, Villringer A, Sacher J (2015) Sex hormones affect neurotransmitters and shape the adult female brain during hormonal transition periods. Front Neurosci 9:37. 10.3389/fnins.2015.00037 25750611 PMC4335177

[CR41] Bellato A, Sesso G, Milone A, Masi G, Cortese S (2024) Systematic review and meta-analysis: altered autonomic functioning in youths with emotional dysregulation. J Am Acad Child Adolesc Psychiatry 63:216–230. 10.1016/j.jaac.2023.01.017 36841327

[CR36] Blaser BL, Weymar M, Wendt J (2023) Associations between fluctuations in premenstrual symptoms and vagally mediated heart rate variability in daily assessments throughout the menstrual cycle: a feasibility study. Authorea Preprints. 10.22541/au.170000985.55376250/v1

[CR14] Brar TK, Singh KD, Kumar A (2015) Effect of different phases of menstrual cycle on heart rate variability (HRV). J Clin Diagn Res 9:CC01–CC04. 10.7860/JCDR/2015/13795.6592 PMC4625231 26557512

[CR12] Cheng YC, Su MI, Liu CW, Huang YC, Huang WL (2022) Heart rate variability in patients with anxiety disorders: a systematic review and meta-analysis. Psychiatry Clin Neurosci 76:292–302. 10.1111/pcn.13356 35340102

[CR2] Comasco E, Kopp Kallner H, Bixo M et al (2021) Ulipristal acetate for treatment of premenstrual dysphoric disorder: a proof-of-concept randomized controlled trial. Am J Psychiatry 178:256–265. 10.1176/appi.ajp.2020.20030286 33297719

[CR39] D’Souza JM, Wardle M, Green CE, Lane SD, Schmitz JM, Vujanovic AA (2019) Resting heart rate variability: exploring associations with symptom severity in adults with substance use disorders and posttraumatic stress. J Dual Diagn 15:2–7. 10.1080/15504263.2018.1526431 PMC6511322 30418104

[CR35] Danel D, Kozak K, Szala A, Kunert-Keil C, Dziedzic-Danel A, Siennicka A (2019) The relationship between the premenstrual syndrome and resting cardiac vagal tone in young healthy females: role of hormonal contraception. Neurophysiology 51:447–454. 10.1007/s11062-020-09841-w

[CR21] de Zambotti M, Nicholas CL, Colrain IM, Trinder JA, Baker FC (2013) Autonomic regulation across phases of the menstrual cycle and sleep stages in women with premenstrual syndrome and healthy controls. Psychoneuroendocrinology 38:2618–2627. 10.1016/j.psyneuen.2013.06.005 23850226 PMC3812396

[CR40] Del Río MI, Molina N, Serrano FG, Molina S, Vigil P (2018) Steroid hormones and their action in women's brains: the importance of hormonal balance. Front Public Health 6:141. 10.3389/fpubh.2018.00141 29876339 PMC5974145

[CR28] Endicott J, Nee J, Harrison W (2006) Daily Record of Severity of Problems (DRSP): reliability and validity. Arch Women Ment Health 9:41–49. 10.1007/s00737-005-0103-y 16172836

[CR10] Fan J, Mei J, Yang Y et al (2024) Sleep-phasic heart rate variability predicts stress severity: building a machine learning-based stress prediction model. Stress Health 40:e3386. 10.1002/smi.3386 38411360

[CR29] Feingold KR, Anawalt B, Blackman MR et al (2017) Diagnostic criteria for premenstrual dysphoric disorder (PMDD). In: Endotext [Internet]. MDText.com, Inc., South Dartmouth, MA. https://www.ncbi.nlm.nih.gov/books/NBK279045/table/premenstrual-syndrom.table1diag/. Accessed 24 Jun 2026

[CR23] Georgiou K, Larentzakis AV, Khamis NN, Alsuhaibani GI, Alaska YA, Giallafos EJ (2018) Can wearable devices accurately measure heart rate variability? A systematic review. Folia Med (Plovdiv) 60:7–20. 10.2478/folmed-2018-0012 29668452

[CR42] Goldberger JJ, Challapalli S, Tung R, Parker MA, Kadish AH (2001) Relationship of heart rate variability to parasympathetic effect. Circulation 103:1977–1983. 10.1161/01.CIR.103.15.1977 11306527

[CR25] Guo Y, Wang H, Zhang H et al (2021) Photoplethysmography-based machine learning approaches for atrial fibrillation prediction: a report from the Huawei Heart Study. JACC Asia 1:399–408. 10.1016/j.jacasi.2021.09.004 36341222 PMC9627828

[CR11] Hartmann R, Schmidt FM, Sander C, Hegerl U (2018) Heart rate variability as indicator of clinical state in depression. Front Psychiatry 9:735. 10.3389/fpsyt.2018.00735 30705641 PMC6344433

[CR43] Hayano J, Yuda E (2021) Assessment of autonomic function by long-term heart rate variability: beyond the classical framework of LF and HF measurements. J Physiol Anthropol 40:21. 10.1186/s40101-021-00272-y 34847967 PMC8630879

[CR9] International Association for Premenstrual Disorders (2023) Premenstrual disorders: facts and figures. https://www.iapmd.org/facts-figures. Accessed 24 Jun 2026

[CR18] Jasinski SR, Presby DM, Grosicki GJ, Capodilupo ER, Lee VH (2024) A novel method for quantifying fluctuations in wearable derived daily cardiovascular parameters across the menstrual cycle. npj Digit Med 7:373. 10.1038/s41746-024-01394-0 39715818 PMC11666598

[CR37] Johnson SC, O’Day J, Kraus E, Delp S, Hicks J (2026) Decoding menstrual health across the lifespan: a scoping review of digital health tools in research. npj Women's Health. 10.1038/s44294-026-00146-7 PMC13549953 42712482

[CR26] Kayacan Y, Makaracı Y, Ozgocer T, Ucar C, Yıldız S (2021) Cortisol awakening response and heart rate variability in the menstrual cycle of sportswomen. Res Q Exerc Sport 92:760–769. 10.1080/02701367.2020.1774486 32853053

[CR22] Kulshreshtha M, Kumar Y, Agarwal V, Dhama V (2013) Symathovagal imbalance in premenstrual syndrome. Indian J Physiol Pharmacol 57:443–447 24968585

[CR19] Landén M, Wennerblom B, Tygesen H et al (2004) Heart rate variability in premenstrual dysphoric disorder. Psychoneuroendocrinology 29:733–740. 10.1016/S0306-4530(03)00117-3 15110922

[CR8] Li Y, Jiang J, Halldorsdottir T et al (2023) Premenstrual disorders and gender differences in adolescent mental health. J Affect Disord 340:930–937. 10.1016/j.jad.2023.08.009 37543115

[CR34] Minson CT, Halliwill JR, Young TM, Joyner MJ (2000) Influence of the menstrual cycle on sympathetic activity, baroreflex sensitivity, and vascular transduction in young women. Circulation 101:862–868. 10.1161/01.CIR.101.8.862 10694525

[CR13] Naim R, Goodwin MS, Dombek K et al (2021) Cardiovascular reactivity as a measure of irritability in a transdiagnostic sample of youth: preliminary associations. Int J Methods Psychiatr Res 30:e1890. 10.1002/mpr.1890 PMC8633925 34390050

[CR7] Opatowski M, Valdimarsdóttir UA, Oberg AS, Bertone-Johnson ER, Lu D (2024) Mortality risk among women with premenstrual disorders in Sweden. JAMA Netw Open 7:e2413394. 10.1001/jamanetworkopen.2024.13394 38805225 PMC11134214

[CR44] Pan Q, Zhou J, Chen M et al (2024) Wearable-measured heart rate variability and premenstrual disorder symptoms across menstrual cycle. medRxiv. 10.1101/2024.10.27.24316196 PMC13342218 42412242

[CR17] Pestana ER, Mostarda CT, Silva-Filho AC, Salvador EP, de Carvalho WRG (2018) Effect of different phases of menstrual cycle in heart rate variability of physically active women. Sport Sci Health 14:297–303. 10.1007/s11332-018-0426-5

[CR45] R Core Team (2023) R: A language and environment for statistical computing. R Foundation for Statistical Computing, Vienna, Austria. https://www.R-project.org

[CR32] Schmalenberger KM, Eisenlohr-Moul TA, Würth L et al (2019) A systematic review and meta-analysis of within-person changes in cardiac vagal activity across the menstrual cycle: implications for female health and future studies. J Clin Med 8:1946. 10.3390/jcm8111946 PMC6912442 31726666

[CR24] Shi X, Chen M, Pan Q et al (2024) Association between dietary patterns and premenstrual disorders: a cross-sectional analysis of 1382 college students in China. Food Funct 15:4170–4179. 10.1039/d3fo05782h 38482855

[CR33] Vongpatanasin W, Tuncel M, Mansour Y, Arbique D, Victor RG (2001) Transdermal estrogen replacement therapy decreases sympathetic activity in postmenopausal women. Circulation 103:2903–2908. 10.1161/01.CIR.103.24.2903 11413078

[CR27] Wu L, He Z, Zhao H et al (2013) Chinese version of Daily Record of Severity of Problems: reliability and validity. J Adv Nurs 69:449–456. 10.1111/j.1365-2648.2012.06070.x 22737971

[CR31] Xia X, Li YH, Liu Y et al (2024) Prevalence of cigarette use and addiction among Chinese females by age and province: findings from nationwide China Health Literacy Survey during 2018–19. Drug Alcohol Depend 258:111258. 10.1016/j.drugalcdep.2024.111258 38503243

[CR6] Yang Q, Þórðardóttir EB, Hauksdóttir A et al (2022) Association between adverse childhood experiences and premenstrual disorders: a cross-sectional analysis of 11,973 women. BMC Med 20:60. 10.1186/s12916-022-02275-7 35184745 PMC8859885

[CR5] Yang Y, Valdimarsdóttir UA, Manson JE et al (2023) Premenstrual disorders, timing of menopause, and severity of vasomotor symptoms. JAMA Netw Open 6:e2334545. 10.1001/jamanetworkopen.2023.34545 37725375 PMC10509727

[CR4] Yang Q, Bränn E, Bertone-Johnson ER et al (2024) The bidirectional association between premenstrual disorders and perinatal depression: a nationwide register-based study from Sweden. PLoS Med 21:e1004363. 10.1371/journal.pmed.1004363 38547436 PMC10978009

[CR16] Yazar Ş, Yazıcı M (2016) Impact of menstrual cycle on cardiac autonomic function assessed by heart rate variability and heart rate recovery. Med Princ Pract 25:374–377. 10.1159/000444322 PMC5588411 26828607

[CR15] Yildirir A, Kabakci G, Akgul E, Tokgozoglu L, Oto A (2001) Effects of menstrual cycle on cardiac autonomic innervation as assessed by heart rate variability. Ann Noninvasive Electrocardiol 7:60–63. 10.1111/j.1542-474X.2001.tb00140.x PMC7027654 11844293

[CR1] Yonkers KA, Simoni MK (2018) Premenstrual disorders. Am J Obstet Gynecol 218:68–74. 10.1016/j.ajog.2017.05.045 28571724

